# Hyper‐IgE syndrome in an 11 year old female presenting with acneiform rash

**DOI:** 10.1002/ski2.297

**Published:** 2023-10-05

**Authors:** Berbie Byrne, Tom Hefferon, Rob Harrington, Marion Leahy, Annette Murphy

**Affiliations:** ^1^ Department of Dermatology University Hospital Galway Galway Ireland

## Abstract

Hyper‐IgE (HIES) is a rare, primary immunodeficiency characterised by eczema, recurrent staphylococcal infections, pneumonia, increased serum IgE and eosinophilia. We present the case of an 11‐year‐old girl presenting to dermatology with an acneiform facial rash and associated bacterial lymphadenitis. History was significant for otitis media, primary tooth retention, low impact wrist fracture, infantile acne and an absence of eczema or pneumonia. Investigations demonstrated mildly elevated IgE, normal eosinophils but positivity for a STAT3 gene mutation—thus representing a case of HIES presenting as an acneiform facial rash with absence of other primary immunological features.

## Graphical abstract

We present the case of an 11 year old girl presenting to dermatology with an acneiform facial rash and associated bacterial lymphadenitis. History was significant for otitis media, primary tooth retention, low impact wrist fracture, infantile acne and an absence of eczema or pneumonia. Investigations demonstrated mildly elevated IgE, normal eosinophils but positivity for a STAT3 gene mutation—thus representing a case of hyper‐IgE presenting as an acneiform facial rash with absence of other primary immunological features.

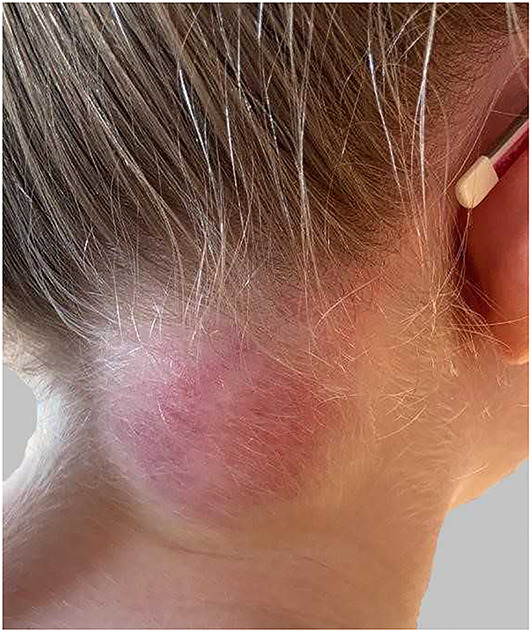

An 11‐year‐old girl was referred with cervical bacterial lymphadenitis thought to be secondary to a progressive acneiform facial rash. Her history was significant for recurrence of bacterial lymphadenitis associated with acne flares over the preceding year.

Her past medical history was significant for infantile acne, recurrent otitis media, post auricular fissuring, primary tooth retention, low impact wrist fracture and recurrent cervical staphylococcus lymphadenitis requiring incision and drainage. There was no significant family history.

Clinically the patient had moderate papulo‐pustular and comedonal acne with coarse skin, multiple atrophic scars, frontal bossing, flat nasal bridge and widening of the nasal tip (Figure 1a). There was a 7 × 7 cm fluctuant swelling on her posterior neck (Figure 1b). She was systemically well and apyrexial.

**FIGURE 1:**
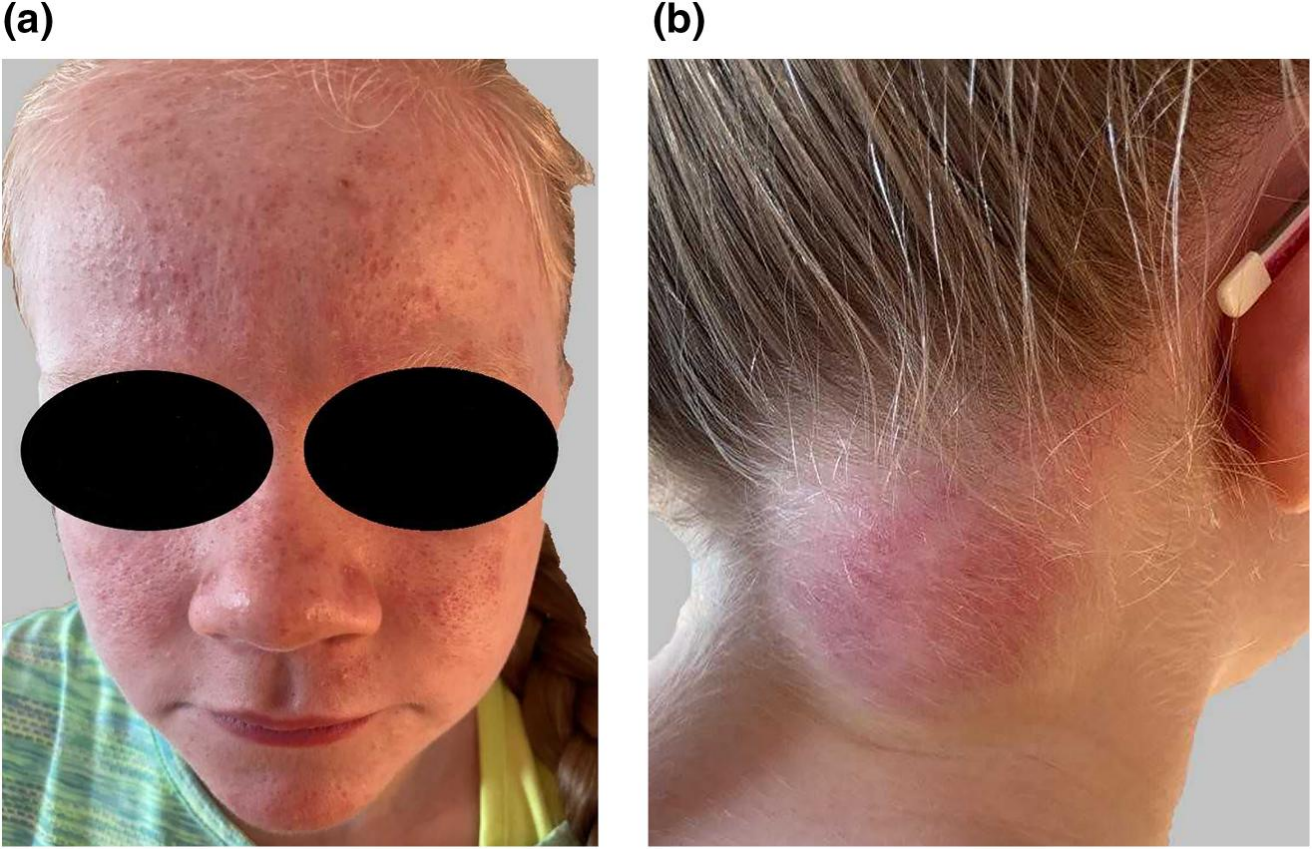
(a) 11 year old girl with papulo‐pustular and comedonal acne, multiple ice pick scars, frontal bossing, flat nasal bridge and widening of the nasal tip. (b) 7 × 7 cm fluctuant mass on the posterior right neck.

Relevant investigations included mild neutropenia (1.1 × 10^9^/l) and mildly elevated IgE (800 IU/mL). Bacterial culture of the cervical lymphadenitis isolated methicillin sensitive *Staphylococcus aureus*. Ultrasound of the neck confirmed right sided necrotic lymph node. The abscess was treated with incision and drainage and intravenous flucloxacillin. Persistent scarring facial acne was subsequently treated with low dose isotretinoin.

Baseline immunology work up is summarised (Table 1). Next generation sequencing to assess for an inborn error of immunity demonstrated a STAT3 gene mutation on chromosome 17q21, consistent with the autosomal or sporadic form of hyper‐IgE (HIES). As there is no family history, this likely represents a de novo mutation.

**TABLE 1:** Baseline immunology investigations.

Investigations	Result
Immunoglobulins IgG, IgM, IgA	Normal
Immunoglobulin IgE	800 (0–56) IU/mL^a^
Eosinophils	Normal
Complement	Normal
Complement classical and alternative pathways	Intact
Lymphocyte subtypes	Normal
Neutrophil oxidative burst test	Normal
Quantiferon	Negative
Vaccination antibody response pneumococcus, tetanus toxoid, haemophilus influenza B	Intact

Abbreviation: HIES, hyper‐IgE.

^a^ Normal reference range for IgE. Levels ≥2000 IU/mL characteristic of HIES.

HIES is a rare, primary immunodeficiency characterised by eczema, recurrent staphylococcal infections, pneumonia, elevated serum IgE (≥2000 IU/mL) and eosinophilia.^1^ Non immunological features include characteristic facies, hyper‐extensibility, low impact fractures, dental abnormalities, retroauricular fissuring, scolioisis and craniosyntosis.^2^ Cutaneous manifestations of HIES include a neonatal papulopustular eruption, generalised eczema, staphylococcal skin infection, coarse facial features and intertriginous folliculitis.^3^ Diagnosis is confirmed by genetic sequencing demonstrating a heterozygous STAT3 gene mutation in the more common sporadic and autosomal dominant HIES. A less common autosomal recessive type involving the DOCK8 gene and other monogenic variants have been identified.

The patient represents an atypical presentation of HIES presenting as an acneiform facial rash in late childhood with an absence of key immunological features. IgE was only mildly elevated and below the threshold level ≥2000 IU/mL for HIES.^4^ The predominant cutaneous feature was localised, scarring facial acne with a notable absence of eczema, folliculitis or staphylococcal skin infection. An acneiform papulo‐pustular eruption frequently misdiagnosed as neonatal acne has been described in neonates with HIES.^5^ Another case of a 13‐year‐old boy presenting with an acneiform rash, orofacial granulomatosis but an absence of the classic triad of HIES features has been described,^6^ with the authors concluding that broad genetic testing strategies will help diagnose patients with an extended phenotype of immunodeficiencies.

The development and progression of our patient's acne occurred simultaneously with the onset and progression of her bacterial cervical lymphadenitis prompting investigation for an underlying immunodeficiency. The milder phenotypic expression of HIES in our patient may account for the later development of her acneiform facial rash and associated bacterial lymphadenitis.

This case highlights the important contribution of genetic analysis in the diagnosis of patients with HIES presenting with a milder disease phenotype. A high clinical index of suspicion in patients with recurrent infection combined with next generation sequencing will aid the future diagnosis of patients presenting with features outside the classic triad of HIES. Cutaneous features remain an important diagnostic clue in the diagnosis of HIES.

## CONFLICT OF INTEREST STATEMENT

None to declare.

## AUTHOR CONTRIBUTIONS

**Berbie Byrne**: Conceptualization (lead); data curation (lead); methodology (lead); project administration (lead); supervision (lead); visualization (lead); writing – original draft (lead); writing – review & editing (lead). **Tom Hefferon**: Data curation (equal); software (equal); visualization (equal). **Rob Harrington**: Software (supporting); visualization (equal); writing – original draft (supporting); writing – review & editing (supporting). **Marion Leahy**: Software (supporting); visualization (supporting); writing – original draft (supporting); writing – review & editing (supporting). **Annette Murphy**: Project administration (supporting); supervision (lead); writing – original draft (equal); writing – review & editing (equal).

## ETHICS STATEMENT

Not applicable.

## Data Availability

The data that support the findings of this study are available from the corresponding author upon reasonable request.

## References

[1] Gharehzadehshirazi A , Amini A , Rezaei N . Hyper IgE syndromes: a clinical approach. Clin Immunol. 2022;237:108988. 10.1016/j.clim.2022.108988 35351598

[2] Grimbacher B , Holland SM , Gallin JI , Greenberg F , Hill SC , Malech HL , et al. Hyper‐IgE syndrome with recurrent infections—an autosomal dominant multisystem disorder. N Engl J Med. 1999;340(9):692–702. 10.1056/nejm199903043400904 10053178

[3] Chamlin SL , McCalmont TH , Cunningham BB , Esterly NB , Lai CH , Mallory SB , et al. Cutaneous manifestations of hyper‐IgE syndrome in infants and children. J Paediatr. 2002;141(4):572–575. 10.1067/mpd.2002.127503 12378200

[4] Erlewyn‐Lajeunesse MD . Hyperimmunoglobulin‐E syndrome with recurrent infection: a review of current opinion and treatment. Pediatr Allergy Immunol. 2000;11(3):133–141. 10.1034/j.1399-3038.2000.00091.x 10981522

[5] Eberting CL , Davis J , Puck JM , Holland SM , Turner ML . Dermatitis and the newborn rash of hyper‐IgE syndrome. Arch Dermatol. 2004;140(9):1119–1125. 10.1001/archderm.140.9.1119 15381553

[6] Carey B , Mercadante V , Fedele S , Glover M , Cale C , Porter S . STAT3‐deficient hyperimmunoglobulin E syndrome: report of a case with orofacial granulomatosis–like disease. Oral Surg Oral Med Oral Pathol Oral Radiol. 2018;126(5):e252–e257. 10.1016/j.oooo.2018.07.006 30126807

